# Generation of a familial hypercholesterolemia model in non-human primate

**DOI:** 10.1038/s41598-023-42763-1

**Published:** 2023-09-20

**Authors:** Akira Sato, Tomoyuki Tsukiyama, Masahiro Komeno, Chizuru Iwatani, Hideaki Tsuchiya, Ikuo Kawamoto, Mitsuru Murase, Takahiro Nakagawa, Iori Itagaki, Yasunari Seita, Shoma Matsumoto, Masataka Nakaya, Akio Shimizu, Atsushi Yamada, Masatsugu Ema, Hisakazu Ogita

**Affiliations:** 1 Division of Molecular Medical Biochemistry, Department of Biochemistry and Molecular Biology, Shiga University of Medical Science, Seta Tsukinowa-Cho, Otsu, Shiga 520-2192 Japan; 2 Research Center for Animal Life Science, Shiga University of Medical Science, Otsu, Japan; 3 Institute for the Advanced Study of Human Biology (WPI-ASHBi), Kyoto University, Kyoto, Japan; 4 Department of Stem Cells and Human Disease Models, Research Center for Animal Life Science, Shiga University of Medical Science, Otsu, Japan; 5 Medical Innovation Research Center, Shiga University of Medical Science, Otsu, Japan

**Keywords:** Biochemistry, Endocrinology

## Abstract

Familial hypercholesterolemia (FH) is an inherited autosomal dominant disorder that is associated with a high plasma level of low-density lipoprotein (LDL) cholesterol, leading to an increased risk of cardiovascular diseases. To develop basic and translational research on FH, we here generated an FH model in a non-human primate (cynomolgus monkeys) by deleting the *LDL receptor* (*LDLR*) gene using the genome editing technique. Six LDLR knockout (KO) monkeys were produced, all of which were confirmed to have mutations in the *LDLR* gene by sequence analysis. The levels of plasma cholesterol and triglyceride were quite high in the monkeys, and were similar to those in FH patients with homozygous mutations in the *LDLR* gene. In addition, periocular xanthoma was observed only 1 year after birth. Lipoprotein profile analysis showed that the plasma very low-density lipoprotein and LDL were elevated, while the plasma high density lipoprotein was decreased in LDLR KO monkeys. The LDLR KO monkeys were also strongly resistant to medications for hypercholesterolemia. Taken together, we successfully generated a non-human primate model of hypercholesterolemia in which the phenotype is similar to that of homozygous FH patients.

## Introduction

Lipid metabolism is tightly regulated, and its dysregulation leads to the progression of several diseases in the cardiovascular system^1,2^. Cholesterol, one of the lipids, plays a role in cell barrier formation, cell membrane fluidity, and signal transduction with other lipids and proteins^3–5^. The main cholesterol carrier in the human body is low-density lipoprotein (LDL)^6,7^. LDL delivers cholesterol to peripheral tissues where LDL is captured via the LDL receptor (LDLR), and is endocytosed into cells by clathrin-mediated internalization^8,9^.

Familial hypercholesterolemia (FH) is an inherited autosomal dominant disorder with high plasma levels of total and LDL cholesterol^10^. The major cause of FH is the mutations in the *LDLR* gene^11^. In most homozygous FH patients who are true homozygotes or compound heterozygotes for two different mutations in the *LDLR* gene, LDLR activity is completely or almost completely lost. These patients show a severely elevated LDL-cholesterol level from birth and often experience fatal cardiovascular disease even in infancy^12^. They do not respond to medications that reduce plasma cholesterol levels, or only do so very poorly, and have a poorer prognosis. It is thus important to discover novel effective medicines and medical treatments for homozygous FH patients.

Many studies using murine hyperlipidemia models have attempted to elucidate the progression of atherosclerosis^13^. However, mice possess the different lipid metabolism system from humans. Loss of LDLR function in mice hardly induces atherosclerosis when the mice are fed with a normal diet^14^. Instead, deficiency of the apolipoprotein E (*Apoe*) gene leads to atherosclerosis even in normal diet-fed mice^15^. On the basis of this, several studies have attempted to use non-murine animals for developing experimental animal models of hyperlipidemia^16–18^. However, to date, no useful FH model has been established in non-human primates.

In this study, we attempted to generate a FH model in non-human primate, cynomolgus monkeys, because among the animals, the lipid metabolism in monkeys is most similar to that in humans^19^. We abolished the expression of LDLR throughout the body of monkeys by the CRISPR/Cas9 genome editing technique. In all of the monkeys, the *LDLR* gene was successfully knocked out, and LDLR expression was not detected. The monkeys showed extremely elevated plasma cholesterol levels that resembled those in homozygous FH patients. Periocular xanthoma was observed in 1-year-old LDLR knockout (KO) monkeys. The monkeys’ responses to some medications for hyperlipidemia were also quite poor.

## Results

### Selection of appropriate guide RNAs to efficiently induce mutations in the monkey *LDLR* gene

**Figure 1:**
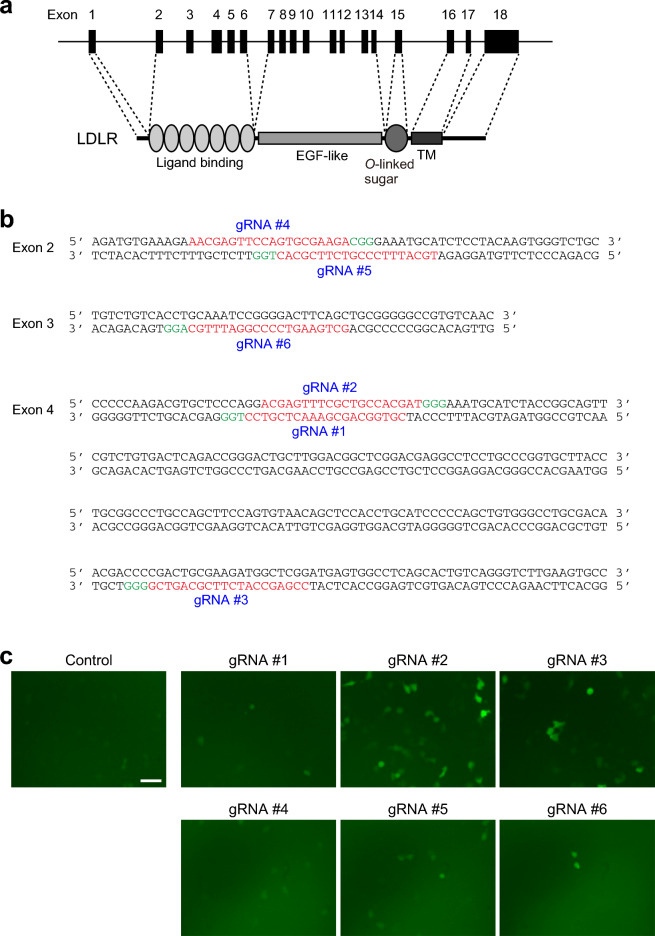
A schematic diagram of targeted sites in the monkey *LDLR* gene. (**a**) The monkey *LDLR* gene located on chromosome 19 is indicated with the number of exons. Representative domains in the LDLR protein are shown below. EGF: epidermal growth factor, TM: transmembrane. (**b**) The monkey *LDLR* gene sequence in exon 2, exon 3, and exon 4. Red: target sequence of each gRNA, Green: protospacer adjacent motif sequence. (**c**) Representative images of HEK293 cells introduced with both pCAG-EGxxFP vector containing the genome fragment including each gRNA target sequence and px459 version 2 vector expressing each gRNA and Cas9 protein. Scale bar: 50 μm.

We aimed to introduce mutations into the *LDLR* gene to abolish its expression throughout the monkey body using the CRISPR/Cas9 genome editing system. To do this, a mixture of guide RNA (gRNA) and recombinant Cas9 protein, called the RNP complex, was injected into metaphase II (MII)-stage oocytes simultaneously with sperm. On the basis of the manufacturer’s algorithm, we designed six gRNAs to target the sequences on exons 2, 3, and 4 of the *LDLR* gene located on chromosome 19 of the monkey genome (Fig. 1a and b). To evaluate the efficiency of the Cas9-mediated digestion conducted by each gRNA, we used the pCAG-EGxxFP vector containing the genome fragment, including each gRNA target sequence, and the px459 version 2 vector expressing both gRNA and Cas9 protein. After both vectors had been transfected into HEK293 cells, we examined the proportion of cells expressing EGFP. EGFP expression was detected when the EGFP cassette in the pCAG-EGxxFP vector was reconstituted by the homology-dependent repair or single-strand annealing of gRNA- and Cas9-mediated digestion of the target sequence. In this single-strand annealing (SSA) assay, gRNA #2 most highly induced EGFP expression among the gRNAs examined, and gRNAs #3 and #5 also showed higher EGFP expression than the control (Fig. 1c). Thus, we selected these three gRNAs (gRNAs #2 and #3 targeting exon 4 and gRNA #5 targeting exon 2) for the following experiments.

The RNP complex, including mixture of the above three gRNAs, was then injected with sperm into MII oocytes. After the injection, the fertilized eggs were incubated for 8 days to develop into blastocysts. We extracted genomic DNA from six independent blastocysts. The exon 2 or exon 4 DNA fragment in the genome was amplified by PCR, and cloned into the pBluescript vector to check the sequence. In contrast to the findings in the in vitro SSA assay, gRNA #2 did not frequently induce mutations in the target sequences, while gRNAs #3 and #5 highly induced mutations, including nucleotide insertion and deletion in and around the target sequences (Table 1). On the basis of this experiment, we decided to use gRNAs #3 and #5 for further experiments to generate LDLR KO cynomolgus monkeys.

**Table 1:** Type and frequency of mutations in the *LDLR* gene of monkey blastocysts by CRISPR/Cas9 genome editing.

	Type of mutations caused by		
	gRNA #2 for exon 4	gRNA #3 for exon 4	gRNA #5 for exon 2
Blastocyst #1	No mutation (100%)	No mutation (14.3%)	2 bp deletion (100%)
		5 bp deletion (85.7%)	
Blastocyst #2	No mutation (100%)	2 bp insertion (20.0%)	16 bp insertion (66.7%)
		6 bp deletion (80.0%)	5 bp deleion (33.3%)
Blastocyst #3	1 bp insertion (80%)	7 bp insertion (20.0%)	5 bp deletion (50.0%)
	1 bp deletion (20%)	5 bp deletion (80.0%)	30 bp deletion (20.0%)
			90 bp deletion (10.0%)
			No mutation (20.0%)
Blastocyst #4	No mutation (100%)	3 bp deletion (100%)	4 bp deletion (100%)
Blastocyst #5	No mutation (100%)	3 bp deletion (100%)	20 bp insertion (100%)
Blastocyst #6	No mutation (100%)	6 bp deletion (100%)	1 bp nonsense mutation (100%)

The percentage of mutations observed in the *LDLR* gene is indicated in ( ).

### Generation of LDLR KO cynomolgus monkeys

**Figure 2:**
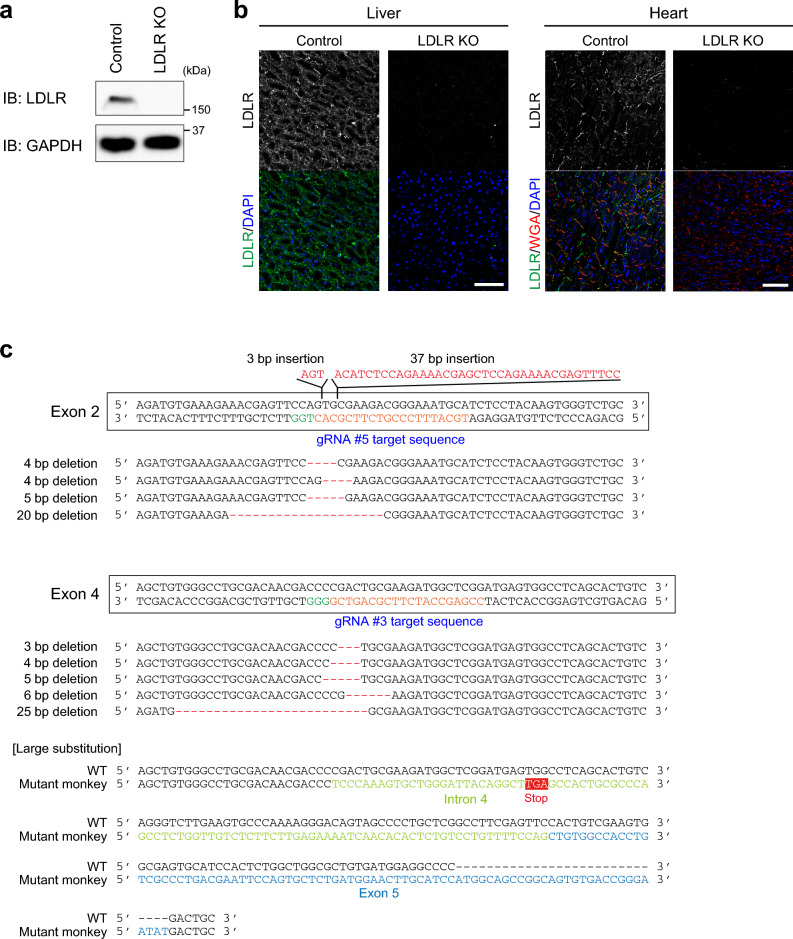
Deletion of LDLR expression in LDLR KO monkey. (**a**) Lysates of the liver isolated from control and LDLR KO monkeys were analyzed by western blotting. GAPDH was blotted as a loading control. (**b**) Immunohistochemical analysis of the liver and heart isolated from control and LDLR KO monkeys with the anti-LDLR antibody. The plasma membrane and nuclei were counterstained with wheat germ agglutinin (WGA) and DAPI, respectively. Scale bars: 100 μm. (**c**) Mutations in the *LDLR* gene of the generated monkeys. Various deletions and insertions in exon 2 and exon 4 were identified by the sequence analysis. In exon 4, a large nucleotide substitution that consisted of a 177 bp sequence including parts of intron 4 and exon 5 was also observed. At the beginning of this substitution, the stop codon (TGA) was occasionally present.

The RNP complex consisting of gRNAs #3 and #5, the recombinant Cas9 protein, and sperm were simultaneously injected into 100 cynomolgus monkey oocytes. After the procedure, 89 in 100 oocytes were alive, and 77 in 89 oocytes were fertilized. Among them, 29 developed into blastocysts, and 27 blastocysts with a healthy appearance were transferred into surrogate mothers. Six monkeys were born. One monkey was not adequately fed by his mother and died just after birth. The other five monkeys developed with normal daily activities. We isolated organs from the dead monkey to examine the expression of LDLR. In western blotting, LDLR expression in the liver was completely absent from this monkey, in contrast to the findings in control wild-type monkey (Fig. 2a). In immunohistochemical analysis, LDLR was clearly expressed in the heart as well as the liver in control monkey, while it was not detected in the organs of the dead monkey (Fig. 2b). Next, we took blood samples from the five surviving 1-year-old monkeys and extracted genomic DNA to examine the mutations in exon 2 and exon 4 of the *LDLR* gene. All of the analyzed genome samples from the five monkeys possessed various deletions and/or insertions of nucleotides in both exon 2 and exon 4 of the *LDLR* gene (Fig. 2c and Table 2). These findings suggest the successful introduction of mutations in the *LDLR* gene that can cause loss of LDLR function in these monkeys.

**Table 2:** Type and frequency of mutations in the *LDLR* gene of blood cells isolated from the generated monkeys.

Identification number [date of birth]	Type of mutations caused by	
	gRNA #3 for exon 4	gRNA #5 for exon 2
#2396 [January 9, 2019]	4 bp deletion (100%)	3 bp insertion (87.5%)
		37 bp insertion (12.5%)
#2397 [January 12, 2019]	5 bp deletion (57.1%)	3 bp insertion (14.3%)
	6 bp deletion (42.9%)	37 bp insertion (85.7%)
#2445 [April 19, 2019]	Large substitution (12.5%)*	4 bp deletion (37.5%)
	3 bp deletion (62.5%)	20 bp deletion (62.5%)
	25 bp deletion (12.5%)	
	No mutation (12.5%)	
#2446 [April 21, 2019]	25 bp deletion (62.5%)	4 bp deletion (100%)
	No mutation (37.5%)	
#2447 [April 24, 2019]	25 bp deletion (12.5%)	5 bp deletion (100%)
	No mutation (87.5%)	

The percentage of mutations observed in the *LDLR* gene is indicated in ( ).

*177 bp sequence including a part of intron 4 and exon 5.

### Phenotypes of LDLR KO cynomolgus monkeys

**Figure 3:**
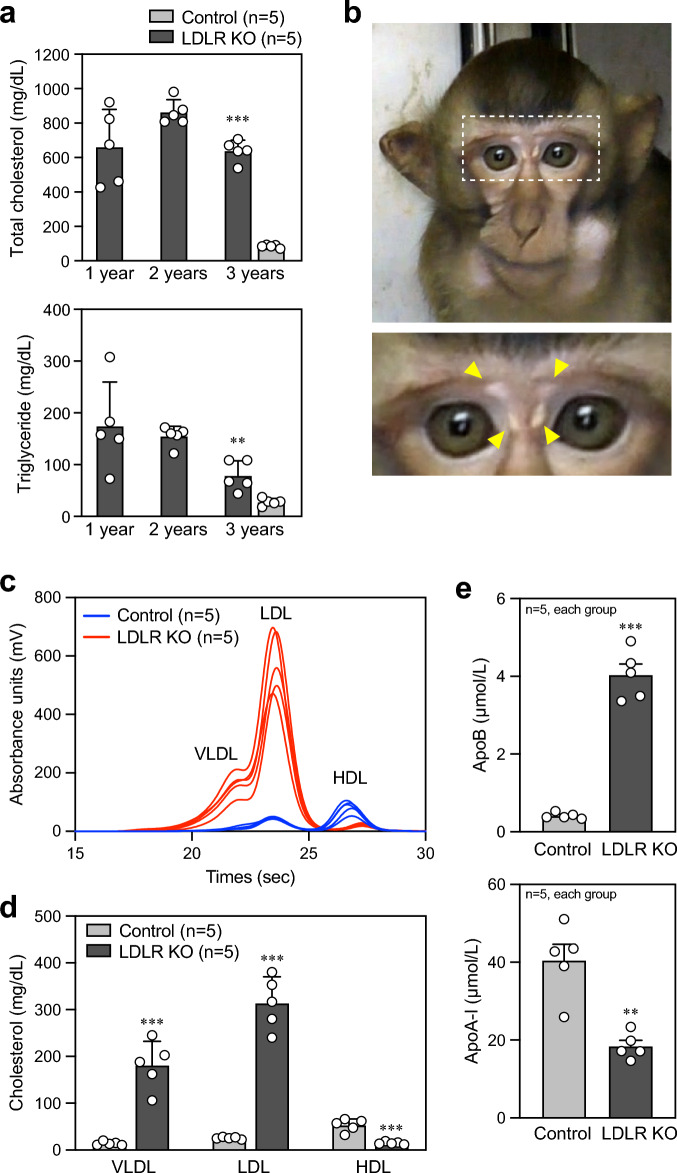
Phenotypes of generated LDLR KO cynomolgus monkeys. (**a**) Total cholesterol and triglyceride concentrations in the plasma isolated from control monkeys at 3 years old and LDLR KO monkeys at 1, 2, and 3 years old. Control: n = 5, LDLR KO: n = 5. **p < 0.01 and ***p < 0.001 vs. control. (**b**) Representative photographs showing face of the LDLR KO monkey. Yellow arrows indicate periocular xanthoma. (**c**) Lipoprotein profile in control (n = 5, blue lines) and LDLR KO (n = 5, red lines) monkeys. The data were obtained from GP-HPLC analysis. (**d**) Graph showing the plasma concentrations of lipoproteins analyzed in (**c**). (**e**) ApoB and apoA-I concentrations calculated by particle numbers of lipoproteins. Control: n = 5, LDLR KO: n = 5. **p < 0.01 and ***p < 0.001 vs. control.

We performed blood tests on control and LDLR KO monkeys. As expected, at 3 years old, all LDLR KO monkeys showed markedly elevated total cholesterol and triglyceride levels in the plasma, compared with control monkeys (Fig. 3a), while liver and renal functions were normal (Table 3). Two of the five LDLR KO monkeys showed periocular xanthoma, which is often observed in FH patients (Fig. 3b). Furthermore, lipoprotein profile analysis was performed, and we found that the plasma LDL- and high-density lipoprotein (HDL)-cholesterol levels were significantly higher and lower, respectively, in LDLR KO monkeys than control monkeys (Fig. 3c and d; Supplementary Table 1). In addition, the plasma concentration of apolipoprotein B (apoB), which is contained in lipoproteins of chylomicron, very low-density lipoprotein (VLDL) and LDL^20^, was increased in LDLR KO monkeys compared with control monkeys, while the concentration of apoA-I, a major protein component of HDL particles^20^, was decreased in LDLR KO monkeys (Fig. 3e). Taken together, these results demonstrate the successful generation of a non-human primate model of hyperlipidemia that resembles the findings in human homozygous FH patients.

**Table 3:** Blood plasma sample data of control and LDLR KO monkeys at 3 years old.

	Control (n = 4)	LDLR KO (n = 5)	Reference value
Total protein (g/dL)	6.5 ± 0.2	6.4 ± 0.2	6.2 ~ 8.4
Albumin (g/dL)	4.2 ± 0.3	4.3 ± 0.2	3.2 ~ 4.8
AST (U/L)	33 ± 4	36 ± 9	23 ~ 92
ALT (U/L)	33 ± 3	33 ± 7	14 ~ 165
γ-GTP (U/L)	65 ± 28	96 ± 32	32 ~ 167
Total bilirubin (mg/dL)	0.3 ± 0.1	0.4 ± 0.1	< 0.5
Creatinine (mg/dL)	0.8 ± 0.1	0.4 ± 0.1	0.6 ~ 1.5
BUN (mg/dL)	17 ± 3	22 ± 14	10 ~ 39
Sodium (mmol/L)	143 ± 1	145 ± 1	143 ~ 155
Potassium (mmol/L)	4.0 ± 0.4	4.2 ± 0.6	3.0 ~ 4.7
Chloride (mmol/L)	105 ± 4	101 ± 2	102 ~ 114
Calcium (mg/dL)	9.0 ± 0.3	9.3 ± 0.3	7.7 ~ 9.7

AST: aspartate aminotransferase, ALT: alanine aminotransferase, γ-GTP: γ-glutamyl transpeptidase, BUN: blood urea nitogen.

### Effects of statins and anti-PCSK9 antibody on LDLR KO monkeys

**Figure 4:**
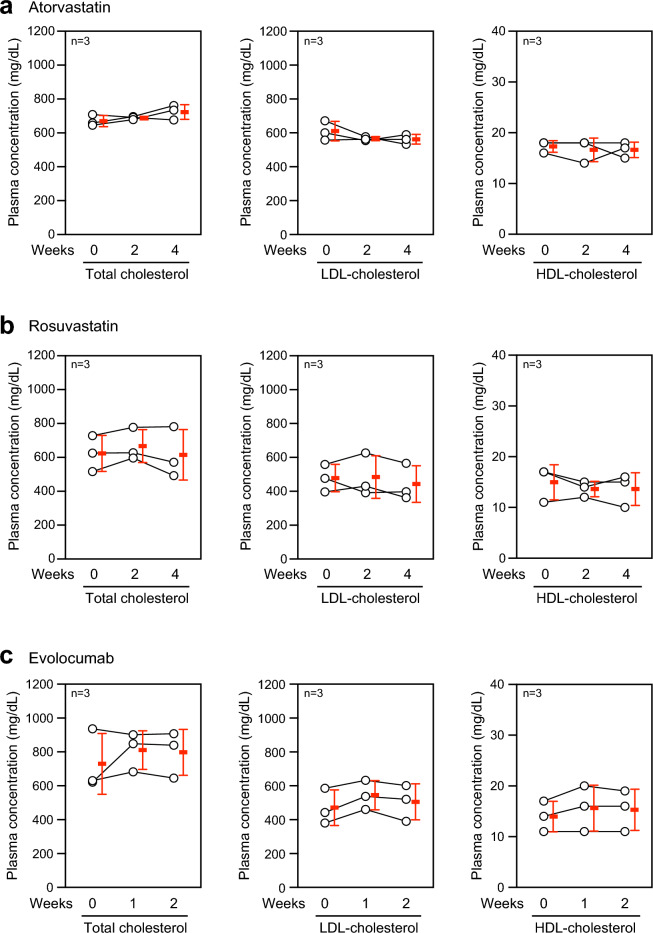
Administration of lipid-lowering drugs in LDLR KO monkeys. ((**a**) and (**b**)) Plasma total, LDL- and HDL-cholesterol concentrations in individual LDLR KO monkeys before (0 week) and 2 and 4 weeks after oral administration of atorvastatin (2 mg/kg/day; (**a**)) or rosuvastatin (1 mg/kg/day; (**b**)) once a day for 4 weeks. (**c**) Plasma total, LDL- and HDL-cholesterol concentrations in individual LDLR KO monkeys before (0 week) and 1 and 2 weeks after single subcutaneous injection of evolocumab (6 mg/kg). n = 3 in each experiment. Red bars indicate mean ± SD.

To examine whether therapeutics for hyperlipidemia can reduce the plasma cholesterol level in LDLR KO monkeys, we first administrated 3-hydroxy-3-methylglutaryl-coenzyme A reductase inhibitors, statins^21^, in LDLR KO monkeys. One of the strong statins, atorvastatin (2 mg/kg/day), was orally administered once a day for 4 weeks. Before and every 2 weeks after the administration, we examined plasma cholesterol levels, and found that this administration did not affect total, LDL-, and HDL-cholesterol levels (Fig. 4a), although it could reduce the total cholesterol level in high fat diet (HFD)-induced hyperlipidemic monkeys (Supplementary Fig. 1). Similar to atorvastatin, rosuvastatin (1 mg/kg/day) administration failed to reduce the plasma cholesterol levels in LDLR KO monkeys (Fig. 4b). Because the doses of atorvastatin and rosuvastatin administered in the monkeys were equivalent to the highest ones for human use, including in homozygous FH patients, the LDLR KO monkeys were considered to be strongly resistant to statins. Next, evolocumab (6 mg/kg), a monoclonal antibody for human PCSK9, was subcutaneously injected into the LDLR monkeys. PCSK9 binds to LDLR to induce the internalization and degradation of this receptor, resulting in decreased expression of LDLR on the surface of cells^22^. If LDLR is still expressed at the minimal level in the LDLR KO monkeys, evolocumab, which inhibits the PCSK9–LDLR binding, would be effective for lowering the plasma cholesterol concentration in the monkeys. We found that the subcutaneous injection of evolocumab did not change plasma cholesterol levels in our LDLR KO monkeys during 2 weeks after the injection (Fig. 4c). Further, we administered probucol (45 mg/kg), which enhances catabolism of LDL independent of the LDL function and increases the excretion of cholesterol into bile^23^, and ezetimibe (0.5 mg/kg), which inhibits gastrointestinal absorption of cholesterol^24^, in LDLR KO monkeys. These lipid-lowering drugs did not decrease the plasma total cholesterol level in the monkeys (Supplementary Fig. 2). Taken together, these results suggest that LDLR function was abolished in the monkeys, and that the CRISPR/Cas9 genome editing system used in this study was effective for ablating the *LDLR* gene.

## Discussion

In this study, we applied a genome editing technique to delete the *LDLR* gene in cynomolgus monkeys and generated a hypercholesterolemia model in non-human primates. To efficiently introduce mutations into the monkey *LDLR* allele by this technique, we established particular settings for the number of gRNAs and the time of gRNA administration in the oocyte. One gRNA is usually administered in the eggs after fertilization has been conducted. In contrast, we injected two gRNAs targeting exon 2 and exon 4 of the *LDLR* gene into the oocyte simultaneously with the sperm in this study. This set-up enabled monkeys with complete loss of LDLR function to be obtained, which is often difficult upon the usage of a single gRNA alone in the CRISPR/Cas9 genome editing for non-human primates^25^. The monkeys presented extremely high levels of plasma cholesterol and were strongly resistant to known medications for treating hyperlipidemia in humans, while control monkeys with HFD responded to atorvastatin. This suggests that the presence of LDLR is required to reduce the plasma cholesterol level by the treatment. The monkey model that we generated resembles homozygous FH patients. In such patients, the condition often results in mortality even in childhood, because it causes severe cardiovascular events and a complete cure is not currently possible. Pharmaceutical companies are continuing to develop more effective therapeutics for homozygous FH patients.

Genetically modified hyperlipidemic mice are useful for research to examine the effect of hyperlipidemia on atherosclerosis and cardiovascular diseases^26,27^. However, it should be kept in mind that lipid metabolism in mice differs from that in humans, resulting in discrepancies in the pathogenesis of such diseases between mice and humans^28^. To overcome this issue, larger animal models, such as rabbits, pigs, and non-human primates, may be helpful, because these animals are more similar to humans anatomically, physiologically, and metabolically^29^. In this context, non-murine animal models for hyperlipidemia were established to examine and reveal the dyslipidemia-induced pathogenesis in humans^30^. Among them, Watanabe heritable hyperlipidemic (WHHL) rabbit, in which natural gene mutations occur, is well known^16^. This rabbit exhibits hyperlipidemia caused by LDLR deficiency that leads to atherosclerosis upon feeding on a normal diet as in FH patients^31,32^. However, there is still a difference in the phenotype caused by LDLR deficiency between rabbits and humans. Because rabbits are herbivores, they are very sensitive to dietary fat. The normal diet for rabbits contains very low level (less than 0.01%) of cholesterol^33^. Heterozygous WHHL rabbits fed with a normal diet show the normal plasma cholesterol level and their LDLR function is not attenuated^34^. However, the diet with only 1% cholesterol can easily increase the concentration of plasma cholesterol in heterozygous WHHL rabbits as well as control New Zealand white rabbits^35^.

Pigs have also been used to generate human-type hyperlipidemia models. Natural mutants harboring both homozygous apoB mutation and homozygous LDLR mutation, which induces R84C amino acid point mutation, were identified to show spontaneous hypercholesterolemia and have atherosclerotic lesions, even when fed with a normal diet^36,37^. Similar to the WHHL rabbit model but in contrast to FH patients, the mutation of the *LDLR* gene in these pigs was recessive. Recently, conventional gene targeting to disrupt the *LDLR* gene in Yucatan miniature pigs and Landrace-Large White crossbred pigs has been performed to produce LDLR KO pigs^17,18^. These animals presented hypercholesterolemia and atherosclerotic lesions when fed with a normal diet, and their atherosclerosis plaque development accelerated under high-fat diet-fed conditions. Similar to the above natural mutant pigs, hyperlipidemia was not observed upon heterozygous *LDLR* gene KO, indicating that the phenotype is expressed in a recessive manner. In contrast to rabbits and pigs, but similar to humans, monkeys with the heterozygous mutation in the *LDLR* gene had the increased level of plasma cholesterol under normal diet-fed condition, and were autosomal dominant inheritance^38^. Taken together with the results in rabbit and pig hyperlipidemia models, non-human primates should be used to generate models that resemble the phenotypes in humans.

Currently, the CRISPR/Cas9-mediated genome editing has been applied in non-human primates, and used to produce several disease models^39–44^. As for lipid metabolism, the genome editing-mediated deletion of PCSK9 in hepatocytes was performed in vivo by using adeno-associated vectors, resulting in efficient reductions of serum PCSK9 and LDL-cholesterol^45^. However, to the best of our knowledge, the generation of a non-human primate model of hyperlipidemia has not previously been reported.

New approaches to develop therapeutics for FH continue in venture enterprises as well as pharmaceutical companies. One of them involves targeting microsomal triglyceride transfer protein (MTP). MTP is localized in the endoplasmic reticulum and transfers triglycerides onto newly synthesized apoB as a critical step in the assembly of chylomicrons in the intestinal enterocytes and VLDL in the hepatocytes^46^. Its deficiency leads to abetalipoproteinemia, an autosomal recessive disease characterized by the absence of plasma lipoproteins and lower plasma concentrations of both triglyceride and cholesterol^47^. On the basis of this, several MTP inhibitors were developed^48,49^. Among them, lomitapide is in clinical use to treat homozygous FH patients. This drug successfully reduces LDL-cholesterol level in homozygous FH patients, although it has adverse effects on the liver^50^. Another newly discovered target for treating homozygous FH patients is angiopoietin-like protein 3 (ANGPTL3). ANGPTL3 is a secreted protein that inhibits lipoprotein lipase, and its deficiency lowers plasma cholesterol level^51,52^. Thus, inhibitors of ANGPTL3, including an inhibitory antibody and vaccination, have been tested in vivo for the treatment of FH^53,54^.

The medications described above are still limited in their ability to ameliorate severe hyperlipidemia in homozygous FH patients, even when administered in combination. Ongoing efforts to find new therapeutics are required to effectively improve the quality of life for homozygous FH patients. Our LDLR KO monkey model could bridge the gap between basic studies and clinical treatment of FH by using it in pre-clinical trials to examine the effect and safety of newly developed medicines on hyperlipidemia.

One of the limitations in this study is that the histological evaluation of atherosclerosis development has not been performed in our LDLR KO monkeys. Due to the young age (approximately 4 years old) of the monkeys, atherosclerosis development is not considered to be high currently. We continue to monitor the monkeys and will check it in the future. Further, we have no data on life expectation and on progression of the disease in the LDLR KO monkeys. Given that the monkeys are still young at this time, it will take several years to obtain the data on these issues.

## Methods

### Study approval

The animal experiments were performed in accordance with the Reporting In Vivo Experiments (ARRIVE) guidelines developed by the National Centre for the Replacement, Refinement & Reduction of Animals in Research (NC3Rs), and also in accordance with The Act on Welfare and Management of Animals from the Japanese Ministry of the Environment, Fundamental Guidelines for Proper Conduct of Animal Experiment and Related Activities in Academic Research Institutions under the jurisdiction of the Japanese Ministry of Education, Culture, Sports, Science and Technology, and Guidelines for Proper Conduct of Animal Experiments from Science Council of Japan. All animal experimental procedures were approved by the Animal Care and Use Committee of Shiga University of Medical Science (approval number: 2017-5-3, 2021-3-11).

### Cynomolgus monkeys

Each cynomolgus monkey was fed with 20 g/kg commercial pellet monkey chow (CMK-1; CLEA Japan, Tokyo, Japan) in the morning, supplemented with 20–50 g of sweet potato in the afternoon^44^. Water was available ad libitum. Temperature and humidity in the animal rooms were maintained at 25 °C and 50%, respectively. The light cycle was 12 h of artificial light from 8 a.m. to 8 p.m. For oocyte collection, female cynomolgus monkeys (*Macaca fascicularis*), ranging in age from 4 to 13 years, were selected for this study^44^. To generate HFD-induced hyperlipidemic monkeys, control monkeys were fed with PS-A pellet monkey chow (Oriental Yeast, Tokyo, Japan) containing saturated fat as palm oil (35% of energy) supplemented with cholesterol at the level of 0.4 mg/kcal. For euthanasia, the monkey was sedated by intramuscular injection of ketamine (5 mg/kg) and xylazine (1 mg/kg), and then pentobarbital sodium (200 mg/kg) was injected intravenously^55^.

### gRNAs

The following sequences are the target sequence of each gRNA in the *LDLR* gene.

1. CGTGGCAGCGAAACTCGTCC.
2. ACGAGTTTCGCTGCCACGAT.
3. CCGAGCCATCTTCGCAGTCG.
4. AACGAGTTCCAGTGCGAAGA.
5. TGCATTTCCCGTCTTCGCAC.
6. GCTGAAGTCCCCGGATTTGC.

Chemically synthesized crRNA targeting each sequence as described above, tracrRNA, and HiFi Cas9 proteins were purchased from Integrated DNA Technologies (Coralville, IA, USA). Each crRNA was incubated with tracrRNA at 95 °C for 5 min and cooled to room temperature to form gRNA. The construction of the gRNA/Cas9 ribonucleoprotein (RNP) complex was performed according to the manufacturer’s instructions.

### Construction of plasmids

pX459 ver.2 (pSpCas9(BB)-2A-Puro V2.0) (Plasmid #62988) and pCAG-EGxxFP (Plasmid #50716) were purchased from Addgene (Watertown, MA, USA)^56^. To construct pX459ver2-*LDLR* gRNA #1–#6, the oligo-DNAs listed below were annealed and ligated into the *Bbs*I site of pX459ver2.

1. 5'-CACCCGTGGCAGCGAAACTCGTCC-3'
  5'-AAACGGACGAGTTTCGCTGCCACG-3'.
2. 5'-CACCACGAGTTTCGCTGCCACGAT-3'
  5'-AAACATCGTGGCAGCGAAACTCGT-3'.
3. 5'-CACCCCGAGCCATCTTCGCAGTCG-3'
  5'-AAACCGACTGCGAAGATGGCTCGG-3'.
4. 5'-CACCAACGAGTTCCAGTGCGAAGA-3'
  5'-AAACTCTTCGCACTGGAACTCGTT-3'.
5. 5'-CACCTGCATTTCCCGTCTTCGCAC-3'
  5'-AAACGTGCGAAGACGGGAAATGCA-3'.
6. 5'-CACCGCTGAAGTCCCCGGATTTGC-3'
  5'-AAACGCAAATCCGGGGACTTCAGC-3'.

To construct pCAG-EGxxFP-*LDLR* exons 2–4, the oligo-DNAs listed below were used in PCR. Amplified PCR products were cloned into *Eco*RI and *Bam*HI sites of pCAG-EGxxFP.

Exon 2: 5'-AAAGGATCCGCTTAATTTCCTGGGAATCAGACTGTTC-3'
  5'-AAAGAATTCGTATCATGCCCAAAGGCGACTCACAGCACG-3'.
Exon 3: 5'-AAAGGATCCCACCATGTTGACATGTTGACCAGGCTGG-3'
  5'-AAAGAATTCACACTTACGACAGTCTTGCTCATCTGATCC-3'.
Exon 4: 5'-CGCGGATCCCCCAAGACGTGCTCCCAGG-3'
  5'-CCGGAATTCGGCAATTCTCCTCGTCAGACTTG-3'.

### The single-strand annealing (SSA) assay

HEK293 cells were purchased from Japanese Collection of Research Bioresources Cell Bank (Osaka, Japan). Cells were transfected with (1) the combination of pX459ver2-*LDLR* gRNA#1, #2, or #3 with pCAG-EGxxFP-*LDLR* exon 4; (2) the combination of pX459ver2-*LDLR* gRNA#4 or #5 with pCAG-EGxxFP-*LDLR* exon 2; and (3) the combination of pX459ver2-*LDLR* gRNA#6 with pCAG-EGxxFP-*LDLR* exon 3. After the transfection using Lipofectamine 2000 (Invitrogen, Waltham, MA, USA), cells were incubated for 2 days in Dulbecco’s Modified Eagle’s Medium (Nacalai Tesque, Kyoto, Japan) containing 10% fetal bovine serum (Sigma-Aldrich, St. Louis, MO, USA). Then, the cells were observed under a fluorescent microscope Eclipse TS100 equipped with Intensilight C-HGF1 (Nikon, Tokyo, Japan).

### Intracytoplasmic injection of both sperm and RNP complex into MII-stage oocytes

Two weeks after the subcutaneous injection of 0.9 mg of a gonadotropin-releasing hormone antagonist (Leuplin for Injection Kit; Takeda Chemical Industries, Osaka, Japan), a microinfusion pump (iPRECIO SMP-200; ALZET Osmotic Pumps, Cupertino, CA, USA) with 15 IU/kg human follicle-stimulating hormone (hFSH, Gonapure Injection; Aska Pharmaceutical, Tokyo, Japan) was embedded subcutaneously under anesthesia and infused at a rate of 7 μL/h for 10 days^44,57^. After the hFSH treatment, 400 IU/kg human chorionic gonadotropin (Gonatropin; Aska Pharmaceutical) was injected intramuscularly. Forty hours later, oocytes were collected by follicular aspiration using a laparoscope (LA-6500; Machida Endoscope, Abiko, Japan). Cumulus-oocyte complexes (COCs) were recovered in Alpha-Modified Eagle’s Medium (MP Biomedicals, Irvine, CA, USA) containing 10% serum substitute supplement (Irvine Scientific, Santa Ana, CA, USA). The COCs were stripped off cumulus cells with 0.5 mg/mL hyaluronidase (Sigma-Aldrich). Fresh sperm were collected by electric stimulation of the penis without anesthesia. Then, a single sperm was captured in the needle attached on the intracytoplasmic sperm injection (ICSI) micromanipulator. This sperm was ejected from the needle into a drop of Opti-MEM (Invitrogen) containing MII oocyte and RNP complexes. Then, the sperm was aspirated again in the needle with the RNP complexes, and both sperm and RNP complexes were co-microinjected into the oocyte. Following the co-injection, embryos were cultured in CMRL Medium-1066 (Invitrogen) supplemented with 20% bovine serum (Invitrogen) at 38 °C in 5% CO_2_ and 5% O_2_.

### DNA sequencing

For DNA sequencing, the PCR products amplified using genomic DNA isolated from blastocysts, blood, and tissue samples were cloned into the *Eco*RV site of pBluescript II vector. The following primers were used for PCR.

Exon 2-F: 5'-CTGTCTCTCTTGGGTGTCTTCCTTGTGT-3'.
Exon 2-R: 5'-ACTTGAGACCAGAAATTCAAGACCAGCA-3'.
Exon 4-F: 5'-ACACCTATTAACGCACCAGTCCTCAGAG-3'.
Exon 4-R: 5'-TCTCCTCGTCAGACTTGTCCTTGCAG-3'.

For each sample, multiple PCR products from different tubes were cloned to reduce bias. After cloning each PCR product into the vector, DNA sequencing using T7 or M13 reverse primer was performed to analyze and determine mutations in each clone.

### Transplantation of embryos into surrogate mothers

When embryos developed to expanded blastocysts, one or two embryos were transferred into appropriate recipient females. Embryos were aspirated into a catheter (ETC3040SM5-17; Kitazato Medical Service, Tokyo, Japan) under a stereomicroscope. The catheter was inserted into the oviduct of the recipient via the fimbria under a laparoscope, and the cultured embryo was transplanted with a small amount of medium^44^. Pregnancy was determined by ultrasonography 30 days after ICSI with the RNP complex.

### Immunofluorescence staining

Cryosections were fixed in 4% paraformaldehyde for 15 min, washed with PBS, and permeabilized with 0.3% Triton X-100 for 5 min^58^. Non-specific staining was reduced by blocking with 3–5% bovine serum albumin (BSA) for 1 h at room temperature. Samples were incubated overnight at 4 °C with anti-LDLR antibody (1:400 dilution) (Abcam, Cambridge, UK). The secondary fluorescent antibodies Alexa Fluor 488 and Alexa Fluor 555 (1:200–1:1000 dilution) (Thermo Fisher Scientific, Waltham, MA, USA) were applied for 1 h at room temperature in the dark. After washing samples with PBS, nuclei were stained with DAPI (1:200 dilution) (Dojindo, Kumamoto, Japan) for 5 min. The cells were viewed using a confocal microscope (FV1000-D; Olympus, Tokyo, Japan, or TCS SP8 X; Leica, Wetzlar, Germany). To visualize the cell membrane, cryosections were fixed in 4% paraformaldehyde, washed with PBS, and incubated with 5 μg/mL Wheat Germ Agglutinin, tetramethylrhodamine conjugate (Invitrogen) in PBS for 8 min. Then, the samples were permeabilized and stained with anti-LDLR antibody.

### Immunoblotting

The liver isolated from a dead monkey was homogenized mechanically in radioimmunoprecipitation assay (RIPA) buffer (50 mmol/L Tris–HCl [pH 7.5], 150 mmol/L NaCl, 0.5% sodium deoxycholate, 0.1% sodium dodecyl sulfate (SDS), 1% Nonidet P-40, 1 μg/mL aprotinin, 1 μg/mL leupeptin, 1 mmol/L phenylmethylsulfonyl fluoride, 5 mmol/L sodium fluoride, and 1 mmol/L sodium orthovanadate)^59^. The lysates were centrifuged at 14,000 rpm for 15 min, and the supernatant was used for further experiments. Protein samples were separated by SDS–polyacrylamide gel electrophoresis (PAGE) and transferred to a polyvinylidene difluoride membrane (Bio-Rad Laboratories, Hercules, CA, USA). The membrane was then blocked for 1 h at room temperature in 5% BSA or 5% skimmed milk in Tris-buffered saline with Tween 20. The membrane was incubated with anti-LDLR antibody (1:1000 dilution) (Abcam) or anti-GAPDH antibody (1:2000 dilution) (Medical & Biological Laboratories, Tokyo, Japan) overnight in 5% skimmed milk at 4 °C, followed by incubation with horseradish peroxidase (HRP)-labeled secondary antibody (GE Healthcare, Piscataway, NJ, USA) for 1 h in 5% skimmed milk. The membrane was incubated with HRP substrate (Luminata Forte; Millipore Corp., Billerica, MA, USA) for 5 min and observed on a luminescent image analyzer LAS-4000 (Fujifilm Life Science, Tokyo, Japan).

### Analysis of blood plasma samples

The blood plasma samples isolated from monkeys were analyzed by VetScan VS2 Chemistry Analyzer (Abaxis, Union City, CA, USA). The reference values were obtained from Tsukuba Primate Research Center for Animal Life Science, National Institutes of Biomedical Innovation, Japan.

### Measurement of cholesterol concentrations

Concentrations of total cholesterol and triglyceride in the plasma isolated from monkeys were measured using the LabAssay Cholesterol kit (Fujifilm Wako, Tokyo, Japan) and the LabAssay Triglyceride kit (Fujifilm Wako) according to the manufacturer’s instructions. For the lipid profile analysis, the blood plasma samples were sent to Immuno-Biological Laboratories (Gunma, Japan), and the gel permeation high-performance liquid chromatography (GP-HPLC) was performed to determine the lipid levels and particle numbers in lipoprotein subclasses. The calculation of plasma apoA-I and apoB concentrations was conducted as previously described^60^. The reference values of lipoproteins in several species were obtained from previous literatures^61–64^.

### Administration of lipid-lowering drugs in monkeys

Oral lipid-lowering drugs were purchased as follows: atorvastatin from Nipro (Osaka, Japan), rosuvastatin from Tokyo Chemical Industry (Tokyo, Japan), probucol from Alfresa Pharma Corp. (Osaka, Japan), and ezetimibe from Nihon Pharmaceutical Industry (Tokyo, Japan). Atorvastatin (2 mg/kg), rosuvastatin (1 mg/kg), probucol (45 mg/kg) or ezetimibe (0.5 mg/kg) was included in a piece of banana that was consumed by the LDLR KO monkeys once a day for 4 weeks. An anti-PCSK9 antibody, evolocumab, was purchased from Amgen (Thousand Oaks, CA, USA). One subcutaneous injection of evolocumab (6 mg/kg) was performed in LDLR KO monkeys. Blood samples were collected from the monkeys before and 2 and 4 weeks after the administration of statins, probucol and ezetimibe, and before and 1 and 2 weeks after the administration of evolocumab. Plasma total cholesterol concentration was measured by the LabAssay Cholesterol kit (Fujifilm Wako), and plasma LDL and HDL concentrations were outsourced to Oriental Yeast.

### Statistics

All data are expressed as the mean ± standard deviation (SD). The statistical differences between experimental groups were evaluated by the two-tailed Student’s *t*-test or a one-way analysis of variance (ANOVA). If ANOVA indicated overall significance, individual differences were evaluated by using the Bonferroni’s post-test. *P* value < 0.05 was considered to be statistically significant.

### Supplementary Information

Supplementary Information.

## Data Availability

The data presented in this article are available from the corresponding author on reasonable request. The sequence data analyzed in this study are available in the European Nucleotide Archive repository, and the study ID for accession is PRJEB58740 (ERP143808).
