# Supplementary material for: Generation of a familial hypercholesterolemia model in non-human primate
Source: Sci Rep. 2023 Sep 20;13:15649. doi: 10.1038/s41598-023-42763-1 (PMC10511719; doi:10.1038/s41598-023-42763-1)
Supplement: Supplementary file 1 — Supplementary Information. [file 41598_2023_42763_MOESM1_ESM.pdf]

Supplementary Information

Generation of a familial hypercholesterolemia model in non-human primate

Akira Sato, Tomoyuki Tsukiyama, Masahiro Komeno, Chizuru Iwatani, Hideaki Tsuchiya, Ikuo Kawamoto, Mitsuru Murase, Takahiro Nakagawa, Iori Itagaki, Yasunari Seita, Shoma Matsumoto, Masataka Nakaya, Akio Shimizu, Atsushi Yamada, Masatsugu Ema, Hisakazu Ogita

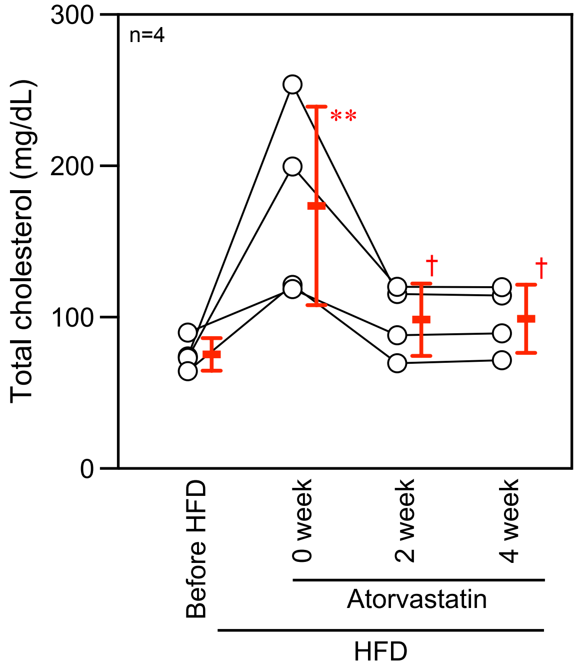

Supplementary Figure 1. Effect of atorvastatin on HFD-induced hypercholesterolemia. Atrorvastatin was administered once a day for 4 weeks in control monkeys fed with HFD. **, $p<0.01$ vs. Before HFD. †, $p<0.05$ vs. 0 week.

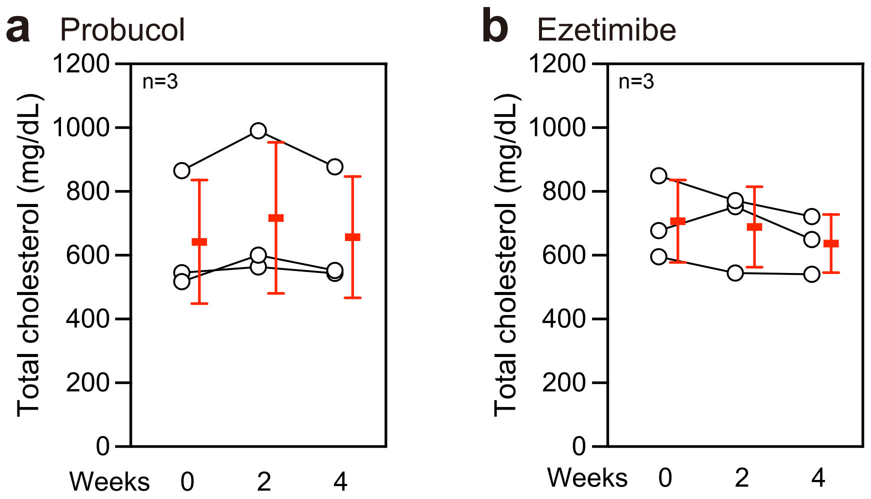

Supplementary Figure 2. Administration of probucol or ezetimibe in LDLR KO monkeys. Plasma total cholesterol concentration in LDLR KO monkeys before (0 week) and 2 and 4 weeks after oral administration of probucol (**a**) or ezetimibe (**b**) once a day for 4 weeks.

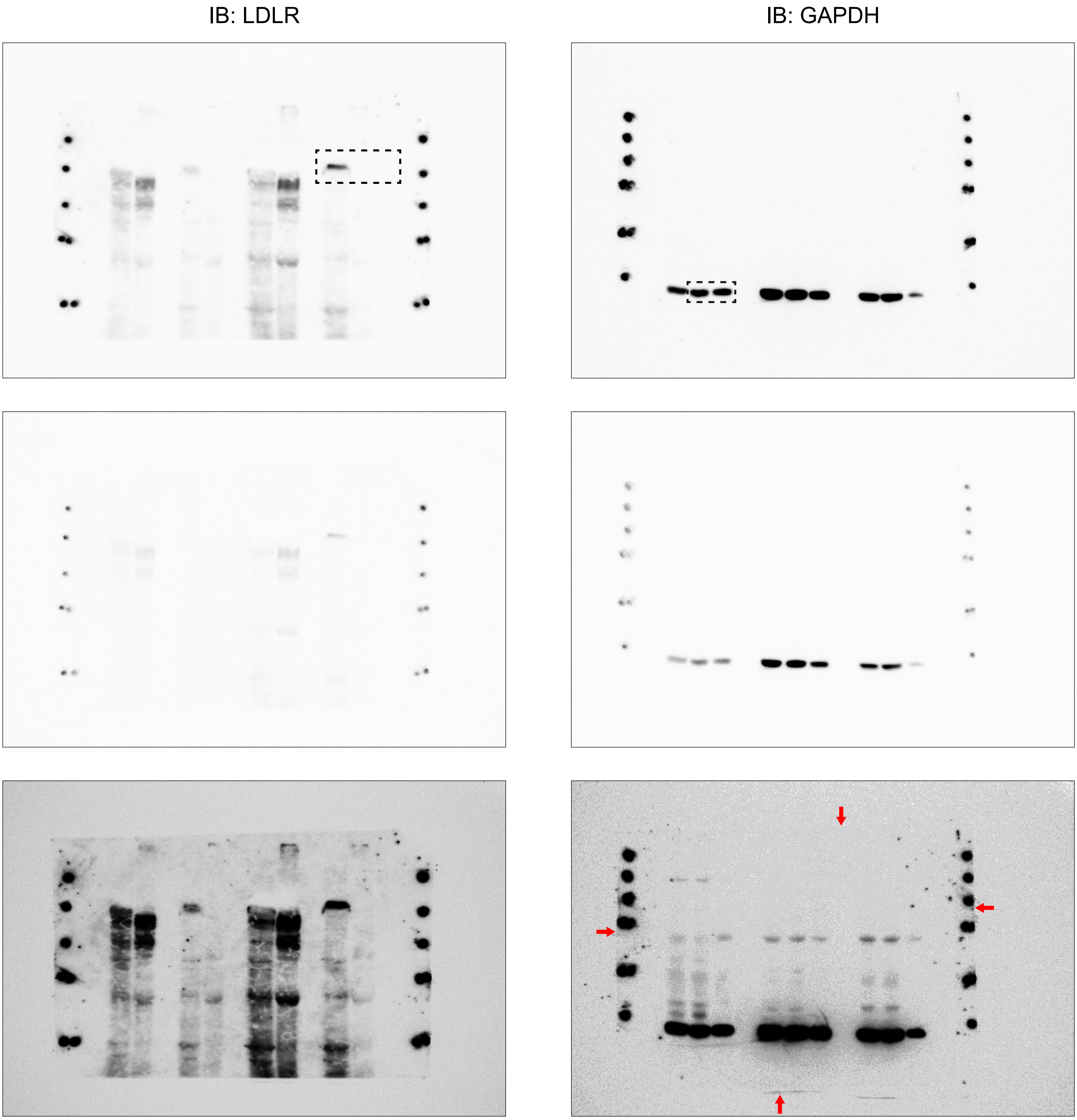

Supplementary Figure 3. Full scan images of immunoblots. Dotted rectangles in the upper images indicate the area presented in Figure 2A. The midle and lower are the low and high exposure images, respectively, of the upper ones. Red arrows indicate the membrane edge.

Supplementary Table 1. Lipid parameters in the plasm of control and LDLR KO monkeys.

		Chylomicron (mg/dL)	VLDL (mg/dL)	LDL (mg/dL)	HDL (mg/dL)
Control (n=5)		0.3 ± 0.1	14.0 ± 3.9	25.4 ± 2.3	52.9 ± 13.4
LDLR KO (n=5)		4.3 ± 3.0*	180.8 ± 51.6***	314.2 ± 55.9***	15.0 ± 2.8***
Reference values	Cynomolgus monkey (Mean age: 6 years)	NR	5 ± 3	34 ± 6	36 ± 3
	Yucatan miniature pig (Mean age: 10 months)	NR	1 ± 1	35 ± 4	33 ± 2
	Beagle dog (Mean age: 5 years)	NR	4 ± 4	19 ± 3	150 ± 31
	Rabbit (Mean age: 9 months)	NR	1 ± 1	5 ± 2	12 ± 5
	Mouse [C57BL/6] (Mean age: 20 weeks)	NR	8 ± 1	21 ± 2	97 ± 4
	Human (Mean age: 61 years)	NR	20 ± 10	93 ± 61	57 ± 17

Data are shown as mean ± SD. NR: not reported. * $p<0.05$ and *** $p<0.001$ vs. Control.
